# Icariside II Enhances Nrf2 Nuclear Translocation to Upregulate Phase II Detoxifying Enzyme Expression Coupled with the ERK, Akt and JNK Signaling Pathways

**DOI:** 10.3390/molecules16119234

**Published:** 2011-11-03

**Authors:** Jing Gu, Xuechao Sun, Guonian Wang, Mingming Li, Meng Chi

**Affiliations:** Department of Anesthesiology, The Third Clinical Hospital, Harbin Medical University, Harbin 150081, China; Email: gujing_1979@hotmail.com (J.G.); sunxuechao@hotmail.com (X.S.); mingminggukuo@126.com (M.L.); chimengzc@hotmail.com (M.C.)

**Keywords:** icariside II, Nrf2, ERK, Akt, JNK, HepG2 cells

## Abstract

In the present study, the potent inducers of phase II detoxifying and antioxidant stress responsive to icariside II was investigated. First, a dose of 0–10 µM icariside II showed no significantly cytotoxicity on HepG2 cells by MTT assays and icariside II could enhance cellular GSH levels by ELISA assay. Then, the potential roles of ERK, Akt and JNK in the regulation of icariside II-induced Nrf2-dependent ARE transcriptional activity as well as ARE-mediated endogenous HO-1 and glutathione GST protein expression in HepG2 cells were estimated. Icariside II activated the nuclear translocation of Nrf2 and the up-regulated expression of Nrf2-related antioxidant protein OH-1 and GST were evaluated by Western blotting. Then the phosphorylation levels of ERK1/2, Akt and JNK1/2 were further examined by Western blotting assays. Results showed that icariside II significantly increased the phosphorylation levels of ERK1/2, Akt and JNK1/2. Furthermore, icariside II-induced ARE transcriptional activity was attenuated by the inhibition of ERK, Akt and JNK signaling using biochemical inhibitors. These results suggest that the Nrf2/ARE pathway plays an important role in the regulation of icariside-mediated antioxidant effects in HepG2 cells.

## Abbreviation

GSH: glutathione
HO-1: heme oxygenase-1
GST: glutathione S-transferase
ARE: antioxidant responsive element
Keap1: Kelch-like ECH-associated protein 1
GCL: g-glutamyl-cysteine ligase
PKC: Protein kinase C
ER: endoplasmic reticulum
VEGF: vascular endothelial growth factor
Glut4: glucose transporter 4

## 1. Introduction

There are many natural and synthetic compounds that can induce phase II detoxifying and antioxidant stress responsive genes. The induction of those genes is a highly effective strategy for protection against antioxidant stress [1,2,3]. The antioxidant responsive element which is located on many phase II/antioxidant genes and binds with the transcription factor Nrf2 is required for the activation of these phase II/antioxidant gene expression induced by compounds. Most recent ﬁndings suggest and support the key role of ARE in the regulation of expression of some phase II and antioxidant stress genes, such as HO-1, GST, and NAD(P)H:quinine oxidoreductase 1 (NQO1), by natural compounds [4,5,6,7,8,9]. Recently, several ARE binding proteins have been identiﬁed, including Nrf1, Nrf2, Nrf3 and small Maf proteins. Nrf2 (Transcription factor nuclear factor-erythroid 2-related factor 2) is an important regulator of cellular oxidative stress which belongs to the basic leucine zipper transcription factors [10]. Normally, Nrf2 binds to Keap1 molecules or is retained in the cytoplasm [11], but Nrf2 was split with Keap1 under oxidative stress and other stimulating factors and then accumulates in the nucleus binding to AREs in order to activate transcription of ARE-mediated genes including HO-1, NQO1, GST, and GCL [12,13,14]. How Nrf2 is transcriptionally activated by such diverse chemical compounds is of wide interest. Previously published reports have shown that multiple signaling kinases are involved in the regulation of the ARE-mediated gene expression in a Nrf2-dependent manner [15,16]. For an example, ERK2, ERK5 and JNK1 upregulated the ARE activity [17], in another hand, p38 MAPK could to suppress the activity [18]. PI3K signaling kinase appears to play a role in nuclear translocation of Nrf2 in response to tBHQ-induced oxidative stress [19]. PKC can directly phosphorylate Nrf2 [20]. Furthermore, Nrf2 is directly phosphorylated by PERK in the ER [21,22], so researchers have also been interested in the pathways of regulation of ARE-mediated gene expression in a Nrf2-dependent manner, such as the ERK1/2, Akt, p38 MAP kinase and JNK signaling pathways.

**Figure 1:**
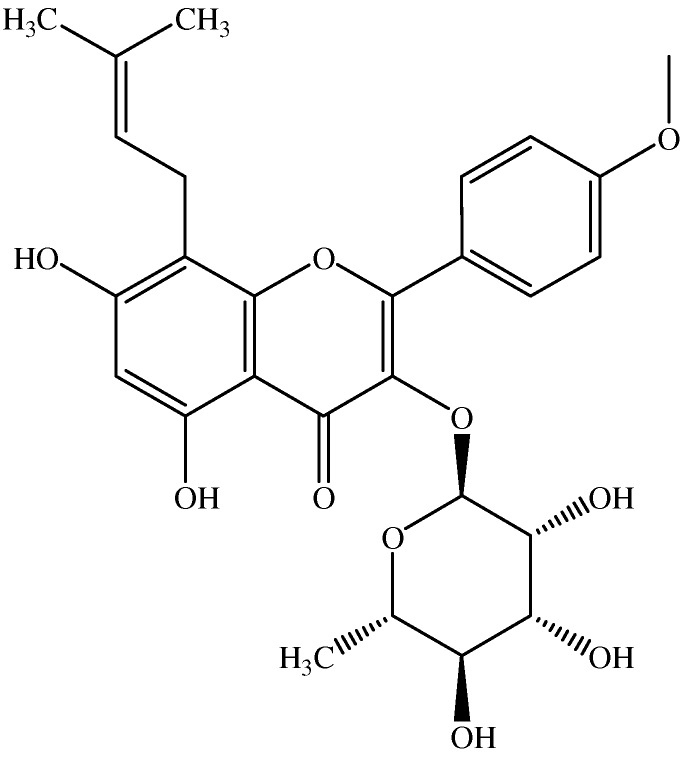
Structure of icariside II.

*Epimedium koreanum* Nakai is a perennial herb that grows in the northeast part of China, north part of North Korea and Japan [23,24]. It is widely used in traditional Chinese and Korean herbal medicine. As a Chinese traditional medicine, it is believed to have various therapeutic properties including those against osteoporosis, erectile function, premature ejaculation, urinary incontinence, joint pain and irregular menstruation [25]. Flavonoids (e.g., icariin) were considered to be the main active components in a previous investigation [26]. Icariside II (Figure 1) is a prenylated active flavonol from the roots of *Epimedium koreanum* Nakai. It was known to decrease level of hypoxia-inducible genes such as VEGF, aldolase A, enolase 1, Glut4 and to induce apoptosis through its anti-inﬂammatory effects in PC-3 prostate cancer cells [27]. To the best of our knowledge, its antioxidant mechanism has not been reported so far. The role of signaling in icariside II-mediated detoxification enzyme expression remains unclear. In the current study, the cytotoxic effect of icariside II was firstly analysis by a MTT assay. Then the Nrf2 nuclear translocation, expression of Nrf2-related antioxidant protein OH-1, GST and the phosphorylation levels of ERK1/2, Akt and JNK1/2 were examined by Western-blot assay. Furthermore, the effects of ERK, Akt, JNK and MAPK signaling inhibitors on expression of antioxidant protein OH-1, GST were also evaluated by Western-blot assay. To the best of our knowledge, this represents the first time the effects of icariside II on Nrf2 nuclear translocation and Phase II Detoxifying Enzyme Expression has been studied.

## 2. Results and Discussion

### 2.1. Cytotoxicity Assays

**Figure 2:**
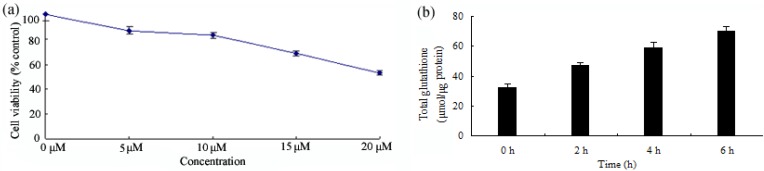
(**a**) Cells were exposed to various concentrations of icariside II for 24 h before being subjected to the MTT assay. Results (presented as mean ± S.D.) are from three independent tests; (**b**) The content of GSH induced by 5 µM icariside II after 0, 2, 4 and 6 h was measured by ELISA and calculated from the average of three independent experiments.

In order to determine the concentration of antioxidant responsive, the cytotoxicity effects of icariside II on HepG2 cells after 24 h exposure were examined (Figure 2a). The relative cell survival rate of HepG2 cells treated with 0–10 µM icariside II was over 80%, as analyzed by a MTT assay, while the survival rates of HepG2 cells treated with 15 and 20 µM icariside II were 69% and 53%, respectively. The results indicate that a dose of 0–10 µM icariside II does not contribute significantly to cytotoxicity in HepG2 cells. GSH is the most powerful intracellular antioxidant and plays a key role in detoxification. Thus, we intended to investigate the effect of icariside II on intracellular glutathione synthesis. Interestingly, icariside II enhanced intracellular GSH levels in a time-dependent manner (Figure 2b). After 5 µM icariside II stimulation for 6 hours, GSH production was about 2.1-fold higher in treated cells than in untreated cells (Figure 2b). The data from the ELISA assay also showed that icariside II could enhance cellular GSH levels. This study has clearly demonstrated that icariside II can promote GSH synthesis, suggesting that icariside II has a cytoprotective role in response to stress.

The anti-cancer capability of icariside II at concentrations above 20 µM was reported [27], which mean the high-does icariside II may display cytotoxicity effects on cells. An antiapoptotic and proapoptotic effect of statins were confirmed [28,29,30,31]; in agreement to previous reports, we supposed icariside II possess both cytotoxic and cytoprotective effects, so the low-dose icariside II (5 µM and 10 µM) was chosen to determine the antioxidant responsive on HepG2 cells.

### 2.2. Icariside II Increased Nrf2 Nuclear Translocation

**Figure 3:**
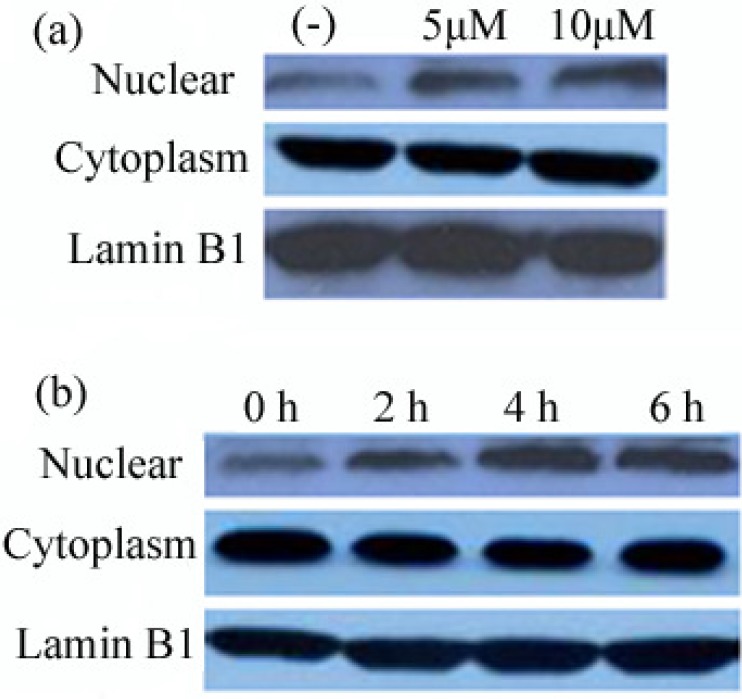
Icariside II stimulates Nrf2 nuclear translocation. Nuclear and cytoplasmic extracts were prepared from HepG2 treated with indicated concentration of icariside II analyzed by Western-blot. (**a**) The levels of Nrf2 in cytoplasmic and nuclear extracts from HepG2 cells treated with 5 and 10 µM Icariside II for 6 h; (**b**) The levels of Nrf2 in cytoplasmic and nuclear extracts from HepG2 cells treated with 5 µM icariside II for 0–6 h.

To investigate the translocation of Nrf2 upon icariside II treatment, the levels of Nrf2 in cytoplasm and nuclear fractions were evaluated by Western-blot analysis in HepG2. As Figure 3a shows, the Nrf2 protein accumulated in the nucleus after exposure to both 5 and 10 μM icariside II for 6 h compared with untreated cells. Five and 10 μM icariside II both induced the accumulation of Nrf2 protein, but no changes in Nrf2 protein concentrations were detected in the nucleus between 5 and 10 μM icariside I, so 5 μM icariside II was chosen for the subsequent investigationd. As shown in Figure 3b, the level of Nrf2 in the nucleus gradually increased with prolonged icariside II treatment.

Nrf2 is a transcription factor expressed in various tissues [32] and cell types [33]. The accumulation of Nrf2 from cytoplasm to nucleus is an essential signaling start for Nrf2-media regulation of antioxidant/detoxification enzymes [34]. As shown in this study, doses of 5 and 10 μM icariside II could induce the nuclear accumulation of Nrf2 in HepG2, that implied icariside II could activate Nrf2 dissociates from Keap 1 (Kelch-like ECH-associated protein 1) and translocates into the nucleus, then increased Nrf2-ARE binding and induce the expression of antioxidant response element (ARE)-regulated genes, such as phase II and stress-responsive antioxidant enzymes. The level of Nrf2 in the nuclear gradually increased with prolonged icariside II treatment implied that Nrf2 might be activated by icariside II to regulate phase II enzyme expression in the time-dependent manner.

### 2.3. Icariside II Induce Nrf2-Related Protein Expression

**Figure 4:**
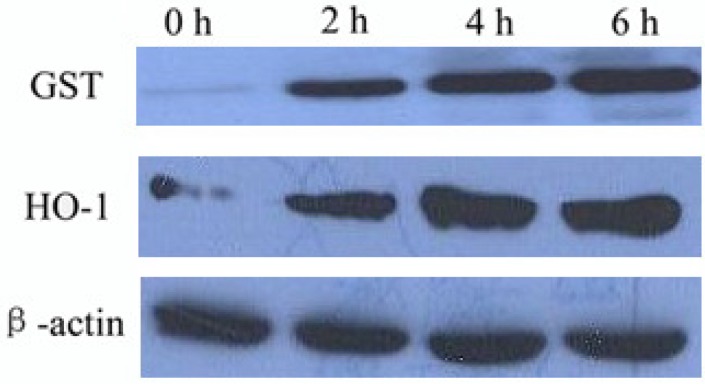
Icariside II (5 μM) induced Nrf2-related antioxidant protein expression in HepG2 were assayed by Western blot analysis.

To investigate the effect of icariside II on the Nrf2-antioxidant system, related phase II antioxidant enzymes were examined by Western-blot [35,36]. As shown in Figure 4, 5 μM icariside II treatment (for 4 h) strongly induced the protein expression of GST and HO-1, two of the phase II antioxidant enzymes in HepG2. Furthermore, the protein expression of GST and HO-1 were the strongest when induced with icariside II for 6 h. The results implied protein expression with icariside II occurs in a time-dependent manner. Based on these results, icariside II could activate phase II antioxidant enzyme expression.

HO-1 protein is an antioxidant enzyme that has been known to be under ARE regulation. HO-1 catalyzes the degradation of heme to CO, iron and biliverdin [12]. It has been implicated to play a protective role in various disease processes such as inflammation, atherosclerosis, and neurodegenerosis [25]. GST protein is another antioxidant enzyme which is under ARE regulation. It was considered to contribute to the phase II biotransformation of xenobiotics by conjugating compounds (often electrophilic and somewhat lipophilic in nature) with reduced glutathione to facilitate dissolution in the aqueous cellular and extracellular media then out of the cell. Based on our results, icariside II induced HepG2 up-regulated the expression of HO-1 and GST related phase II antioxidant enzymes in time-dependent manner (Figure 4). But there was a very interesting result whereby Nrf2 was markedly increased with icariside II treatment at 2 h (Figure 3b), but the obvious expression of HO-1 and GST were shown at 4 h with icariside II treatment, that is, there was a delay in the indiction of the accumulation of HO-1 and GST. It may be suggested that this delay might result from the net effects of transcription factors, such as NF-kB, AP-1, AP-2, and IL-6 response element which can bind to the sites in the promoter region of the HO-1 gene [37]. Taken together with the MTT analysis results, Nrf2 nuclear translocation and Nrf2-related protein expression which were induced by icariside II, this study has demonstrated quite clearly that icariside II can promote Nrf2-ARE binding and activate the phase II antioxidant enzyme expression, suggesting that icariside II has a cytoprotection role in response to stress.

### 2.4. ERK, AKT, and JNK Signaling Pathways Participate in the Icariside II-Induced Phase II Protein Expression

**Figure 5:**
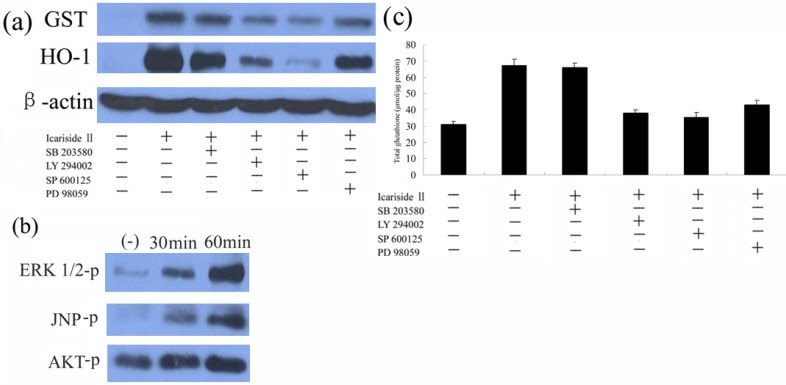
AKT, ERK, and JNK signaling pathways participate in the Icariside II-induced phase II protein expression in HepG2 cells. (**a**) Cells were treated with 10 µM LY294002, SB203580, SP600125, or PD98059 for 30 min and then challenged with 5 µM Icariside II for another 6 hours. Total cell extracts were prepared and subjected to western blot analysis for detection of the levels of GST and HO-1; (**b**) Cells were treated with 5 µM Icariside II for 0–60 min. Total cell extracts were prepared and subjected to western- blot analysis in order to detect the active phosphorylated forms of AKT, ERK1/2 and JNK; (**c**) Cells were treated as described in (A) and ELISA analysis was applied to measure the contents of GSH. Results (presented as mean ± S.D.) are from three independently conducted experiments.

LY294002 (an inhibitor of the PI3K family), SB203580 (an inhibitor of p38 MAP kinase), SP600125 (an inhibitor of JNK1/2), and PD98059 (an inhibitor of MEK1/2) were used in pretreatment of HepG2 cells for 30 min, and then cells were co-treated with 5 µM icariside II for another 6 h, then the levels of GST, and HO-1 were determined by Western-blot. As shown in Figure 5a, LY294002, SP600125, and PD98059 could effectively suppress the up-regulation of these protein expressions by icariside II, while SB203580 failed to repress icariside II-mediated protein expression (Figure 5a).

The cellular biochemical processes are regulated by signaling pathways, including protein localization, responses to stress and so on [30]. Thus, the effect of signaling pathways involved in the regulation of icariside II-mediated Nrf2 activation were evaluated using Western blot analysis. Extracellular signal-regulated phosphorylation of kinase AKT, ERK, and JNK were analysed using antibodies specific to forms of phosphorylated kinases. These results indicate that the AKT, ERK, and JNK signaling pathways might be involved in the regulation of icariside II-mediated phase II enzyme expression. The results presented in Figure 5b revealed that icariside II is able to activate the AKT, ERK, and JNK signaling pathways, in a time-dependent manner (Figure 5b), The overall protein levels of these kinases were similar. It is conceivable that icariside II-enhanced phase II protein expression through AKT, ERK, and JNK signaling pathways regulates the enhancement of Nrf2 activation and translocation.

The PKC, MAPK and PI3K/AKT signaling pathways have been reported to play a distinct role in the activation of Nrf2 [33]. In this study, the expression level of antioxidant enzyme GST and HO-1 (Figure 5a) was specifically suppressed by the addition of inhibitors of the AKT, ERK, and JNK signaling pathways, the GSH generation enhanced by icariside II also was suppressed by the AKT, ERK, and JNK inhibitors (Figure 5c), but not the p38 MAP kinase inhibitor, indicating that the AKT, ERK, and JNK signaling pathways, but not p38 MAP kinase, might be involved in the regulation of icariside II-mediated phase II enzyme expression. Consequently, the extent of AKT, ERK, and JNK phosphorylation following icariside II treatment was further investigated. The results (Figure 5b) revealed that icariside II was able to activate AKT, ERK, and JNK signaling pathways in a time-dependent manner, consistent with the data in Figure 4A. Based on these results, we conclude that AKT, ERK, and JNK signaling pathways are indeed involved in icariside II-mediated Nrf2 activation and involved in phase II enzyme expression.

Icariside II has been reported to induce tumor cell apoptosis through its anti-inﬂammatory effects by ROS-mediation [31] and enhancement of antioxidant response via Nrf2-mediated gene expression in this study. Similarly, cinnamaldehyde possesses both cytotoxic and cytoprotective effects [23]. Mostly, the different concentrations and duration of icariside II would determine the cell fate. Of course, the cytotoxic and cytoprotective effects of icariside II also depend on extracellular stress, the type of cell situation, and the whether it is in the dynamic redox balance, especially since the balance of the signaling and redox state is always disregulated in tumor cells. Therefore, AKT, ERK and JNK signaling pathways provide evidence of icariside II-enhanced phase II protein expression and confirm the cytoprotective effects of icariside II.

## 3. Experimental

### 3.1. Cell Culture

HepG2 (Human hepatocarcinoma) cells obtained from The 2nd Affiliated Hospital of Harbin Medical University, China. All the cells were maintained in RPMI 1640 medium supplemented with 10% fetal bovine serum and 100 U/mL penicillin and 100 g/mL streptomycin. The cells were kept at 37 °C in a humidiﬁed atmosphere containing 5% CO_2_.

### 3.2. Chemicals and Antibodies

Icariside II was purchased from Sigma-Aldrich (St. Louis, MO, USA). PD98059, SB203580, SP600125, and LY294002 were purchased from Tocris Bioscience (Ellisville, MO, USA). Antibodies against phospho-p44/42 MAPK (Thr202/Tyr204) (#9101), phospho-JNK (Thr183/Tyr185) (#9251) and phospho-Akt (Ser473) (D9E) were obtained from Cell Signaling Technology (Beverly, MA, USA). Antibodies against Nrf2 and HO-1, GST, and lamin B1 were obtained from Santa Cruz Biotechnology (Santa Cruz, CA, USA). Mouse monoclonal beta-actin antibody and was from obtained from Chemicon (Temecula, CA, USA). MTT was obtained from Sigma-Aldrich Inc. (St. Louis, MO, USA). Deionized water was used in all experiments.

### 3.3. Viability Assay

Inhibition of cell proliferation by icariside II was measured by the MTT assay [19]. Brieﬂy, HepG2 cells were plated in 96-well culture plates (1 × 10^5^ cells/well) separately. After 24 h incubation, cells were treated with cajanol (0, 5, 10, 15 and 20 μM, eight wells per concentration) for 24 h, MTT solution (5 mg/mL) was then added to each well. After 4 h incubation, the formazan precipitate was dissolved in 100 L dimethyl sulfoxide, and then the absorbance was measured in an ELISA reader (Thermo Molecular Devices Co., Union City, CA, USA) at 570 nm. The cell viability ratio was calculated by the following formula: Inhibitory ratio (%) = [(OD_control_ − OD_treated_)/(OD_control_)] × 100%.

### 3.4. Determination of Intracellular GSH Levels

For measurement of intracellular GSH levels, ELISA analysis was performed as previously described [38]. HepG2 cells were treated with 5 µM icariside II for 0, 2, 4 and 6 h, and then stained with 50 µM chloromethylfluorescein diacetate (CMF-DA) for an additional 30 min. The stained cells were analyzed immediately by a MicroplateReader (RMR-950A, Risingmed) using a wavelength of 350 nm. The results are expressed as nmol GSH/mg of protein.

### 3.5. Western-Blot Analysis

For isolation of protein fractions, HepG2 cells (1 × 10^6^) were seeded in a 6 cm plate and incubated at 37 °C for 24 h. Cells were treated with various concentrations of icariside II for 0–6 h and then washed twice with ice-cold PBS, and lysed using cell lysis buffer [20 mM Tris pH 7.5, 150 mM NaCl, 1% Triton X-100, 2.5 mM sodium pyrophosphate, 1 mM EDTA, 1% Na3CO4, 0.5 g/mL leupeptin, 1 mM phenylmethanesulfonyl ﬂuoride (PMSF)]. The lysates were collected by scraping from the plates and then centrifuged at 10,000 rpm at 4 °C for 5 min. The protein samples (20 g) were loaded on a 12% of SDS-polyacrylamide gel for electrophoresis, and transferred onto PVDF transfer membranes (Millipore, Billerica, MA, USA) at 0.8 mA/cm2 for 2 h. Membranes were blocked at room temperature for 2 h with blocking solution (1% BSA in PBS plus 0.05% Tween-20). Membranes were incubated overnight at 4 °C with primary anti-bodies at a 1:1000 dilution (Biosynthesis Biotechnology Company, Beijing, China) in blocking solution. After thrice washings in TBST for each 5 min, membranes were incubated for 1 h at room temperature with an alkaline phosphatase peroxidase-conjugated anti-mouse secondary antibody at a dilution of 1:500 in blocking solution. Detection was performed by the BCIP/NBT Alkaline Phosphatase Color Development Kit (Beyotime Institute of Biotechnology, Suzhou, China) according to the manufacturer’s instructions. Bands were recorded by a digital camera (Canon, EOS 350D, Tokyo, Japan).

### 3.6. Statistical Analyses

Data are presented as means ± SEM. Differences were evaluated using unpaired Student’s t-tests. The level of statistical signiﬁcance was set at *p* < 0.05.

## 4. Conclusions

In several diseases, such as ischemia-reperfusion injury, major burns and pyaemia caused by oxidative stress in cardiovascular and transplant surgery, free radical-mediated oxidative stress play an important clinical role, which causes the depletion of the body’s antioxidant defense system. Antioxidant supplements enhance the body’s antioxidant defense capacity and reduce the resulting tissue damage. The present study indicates that icariside II expresses antioxidant effects in HepG2 as a result of inducing antioxidant enzymes via the Nrf2/ARE pathway. The study provides evidence of a protective role for icariside II against oxidative injury and further indicates the multiple effects of icariside II, which could potentially contribute to antioxidant protection.

## References

[1] Chen C., Kong A.N. (2005). Dietary cancer-chemopreventive compounds: From signaling and gene expression to pharmacological effects. Trends Pharmacol. Sci..

[2] Kong A.N. (2004). Signal transduction in cancer chemoprevention. Mutat. Res..

[3] Yu X., Kensler T. (2005). Nrf2 as a target for cancer chemoprevention. Mutat. Res..

[4] Kong A.N., Owuor E., Yu R. (2001). Induction of xenobiotic enzymes by the MAP kinase pathway and the antioxidant or electrophile response element (ARE/EpRE). Drug Metab. Rev..

[5] Keum Y.S., Owuor E.D., Kim B.R., Hu R., Kong A.N. (2003). Involvement of Nrf2 and JNK1 in the activation of antioxidant responsive element (ARE) by chemopreventive agent phenethyl isothiocyanate (PEITC). Pharm. Res..

[6] Rushmore T.H., Kong A.N. (2002). Pharmacogenomics, regulation and signaling pathways of phase I and II drug metabolizing enzymes. Curr. Drug Metab..

[7] Chen C., Pung D., Leong V. (2004). Induction of detoxifying enzymes by garlic organosulfur compounds through transcription factor Nrf2: Effect of chemical structure and stress signals. Free Radic. Biol. Med..

[8] Hu R., Hebbar V., Kim B.R. (2004). *In vivo* pharmacokinetics and regulation of gene expression profiles by isothiocyanate sulforaphane in the rat. J. Pharmacol. Exp. Ther..

[9] Itoh K., Chiba T., Takahashi S. (1997). An Nrf2/small Maf heterodimer mediates the induction of phase II detoxifying enzyme genes through antioxidant response elements. Biochem. Biophys. Res. Commun..

[10] Itoh K., Wakabayashi N., Katoh Y. (1999). Keap1 represses nuclear activation of antioxidant responsive elements by Nrf2 through binding to the amino-terminal Neh2 domain. Genes Dev..

[11] Forman H.J., Ruden D. (2004). Introduction to serial reviews on EpRE and its signaling pathway. Free Radic. Biol. Med..

[12] Jaiswal A.K. (2004). Nrf2 signaling in coordinated activation of antioxidant gene expression. Free Radic. Biol. Med..

[13] Itoh K., Tong K.I., Yamamoto M. (2004). Molecular mechanism activating Nrf2-Keap1 pathway in regulation of adaptive response to electrophiles. Free Radic. Biol. Med..

[14] Nguyen T., Yang C.S., Pickett C.B. (2004). The pathways and molecular mechanisms regulating Nrf2 activation in response to chemical stress. Free Radic. Biol. Med..

[15] Chen C., Yu R., Owuor E.D., Kong A.N. (2000). Activation of antioxidant-response element (ARE), mitogen-activated protein kinases (MAPKs) and caspases by major green tea polyphenol components during cell survival and death. Arch. Pharm. Res..

[16] Shen G., Hebbar V., Nair S. (2004). Regulation of Nrf2 transactivation domain activity. The differential effects of mitogen-activated protein kinase cascades and synergistic stimulatory effect of Raf and CREB-binding protein. J. Biol. Chem..

[17] Yu R., Lei W., Mandlekar S. (1999). Role of a mitogen-activated protein kinase pathway in the induction of phase II detoxifying enzymes by chemicals. J. Biol. Chem..

[18] Yu R., Mandlekar S., Lei W., Fahl W.E., Tan T.H., Kong A.T. (2000). p38 mitogen-activated protein kinase negatively regulates the induction of phase II drug-metabolizing enzymes that detoxify carcinogens. J. Biol. Chem..

[19] Kang K.W., Lee S.J., Park J.W., Kim S.G. (2002). Phosphatidylinositol 3-kinase regulates nuclear translocation of NF-E2-related factor 2 through actin rearrangement in response to oxidative stress. Mol. Pharmacol..

[20] Huang H.C., Nguyen T., Pickett C.B. (2002). Phosphorylation of Nrf2 at Ser-40 by protein kinase C regulates antioxidant response element-mediated transcription. J. Biol. Chem..

[21] Cullinan S.B., Zhang D., Hannink M., Arvisais E., Kaufman R.J., Diehl J.A. (2003). Nrf2 is a direct PERK substrate and effector of PERK-dependent cell survival. Mol. Cell. Biol..

[22] Cullinan S.B., Diehl J.A. (2004). PERK-dependent activation of Nrf2 contributes to redox homeostasis and cell survival following endoplasmic reticulum stress. J. Biol. Chem..

[23] Kee C.H. (2000). The Pharmacology of Chinese Herbs.

[24] Oh M.H., Houghton P.J., Whang W.K., Cho J.H. (2004). Screening of Korean herbal medicines used to improve cognitive function for anti-cholinesterase activity. Phytomedicine.

[25] Meng F.H., Li Y.B., Xiong Z.L., Jiang Z.M., Li F.M. (2005). Osteoblastic proliferative activity of *Epimedium brevicornum* Maxim. Phytomedicine.

[26] Li W.K., Zhang R.Y., Xiao P.G. (1996). Flavonoids from *Epimedium wanshanense*. Phytochemistry.

[27] Lee K.S., Lee H.J., Kwang S.A., Kim S.H., Nam D., Kim D.K., Choi D.Y., Ahn K.S., Lu J., Kim S.H. (2009). Cyclooxygenase-2/prostaglandin E 2 pathway mediates icariside II induced apoptosis in human PC-3 prostate cancer cells. Cancer Lett..

[28] Kureishi Y., Luo Z., Shiojima I. (2000). The HMG-CoA reductase inhibitor simvastatin activates the protein kinase Akt and promotes angiogenesis in normocholes-terolemic animals. Nat. Med..

[29] Miyashita Y., Ozaki H., Koide N. (2002). Oxysterol-induced apoptosis of vascular smooth muscle cells is reduced by HMG-CoA reductase inhibitor, pravastatin. J. Atheroscler. Thromb..

[30] Son B.K., Kozaki K., Iijima K. (2006). Statins protect human aortic smooth muscle cells from inorganic phosphate-induced calcification by restoring Gas6-Axl survival pathway. Circ. Res..

[31] Guijarro C., Blanco-Colio L.M., Ortego M. (1998). 3-Hydroxy-3 methylglutaryl coenzyme A reductase and isoprenylation inhibitors induce apoptosis of vascular smooth muscle cells in culture. Circ. Res..

[32] Lee T.D., Yang H., Whang J., Lu S.C. (2005). Cloning and characterization of the human glutathione synthetase 5′-flanking region. Biochem. J..

[33] Pi J., Bai Y., Reece J.M., Williams J., Liu D., Freeman M.L., Fahl W.E., Shugar D., Liu J., Qu W., Collins S., Waalkes M.P. (2007). Molecular mechanism of human Nrf2 activation and degradation: role of sequential phosphorylation by protein kinase CK2. Free Radic. Biol. Med..

[34] Nguyen T., Yang C.S., Pickett C.B. (2004). The pathways and molecular mechanisms regulating Nrf2 activation in response to chemical stress. Free Radic. Biol. Med..

[35] Li J., Ichikawa T., Janicki J.S., Cui T. (2009). Targeting the Nrf2 pathway against cardiovascular disease. Expert Opin. Ther. Targets.

[36] Warabi E., Takabe W., Minami T. (2007). Shear stress stabilizes NF-E2-related factor 2 and induces antioxidant genes in endothelial cells: Role of reactive oxygen/nitrogen species. Free Radic. Biol. Med..

[37] Haridas M., Hanausek G., Nishimura H., Soehnge W., Zbigniew M., Narog E., Spears R., Gutterman J.U. (2004). Triterpenoid electrophiles (avicins) activate innate stress response by redox regulation of a gene battery. J. Clin. Invest..

[38] Wasserman W.W., Fahl W.E. (1997). Functional antioxidant responsive elements. Proc. Natl. Acad. Sci. USA.

